# Effect of hyperlipidemia on the expression of circadian genes in apolipoprotein E knock-out atherosclerotic mice

**DOI:** 10.1186/1476-511X-8-60

**Published:** 2009-12-30

**Authors:** Likun Hou, Chao Lu, Yang Huang, Sifeng Chen, Luchun Hua, Ruizhe Qian

**Affiliations:** 1 Department of Physiology and Pathophysiology, Fudan University Shanghai Medical College, Shanghai 200032, PR China; 2 Department of Surgery, Huashan Hospital affiliated to Fudan University, Shanghai 200040, PR China; 3 Department of Pathology, Shanghai Pulmonary Hospital affiliated to Tongji University, Shanghai 200433, PR China

## Abstract

**Background:**

Circadian patterns of cardiovascular vulnerability were well characterized, with a peak incidence of acute myocardial infarction and stroke secondary to atherosclerosis in the morning, which showed the circadian clock may take part in the pathological process of atherosclerosis induced by hyperlipidemia. Hence, the effect of hyperlipidemia on the expression of circadian genes was investigated in atherosclerotic mouse model.

**Results:**

In apoE-/-mice on regular chow or high-fat diet, an atherosclerotic mouse model induced by heperlipidemia, we found that the peak concentration of serum lipids was showed four or eight hours later in apoE-/- mice, compared to C57BL/6J mice. During the artificial light period, a reduce in circulating level of serum lipids corresponded with the observed increase of the expression levels of some the transcription factors involved in lipid metabolism, such as PPARα and RXRα. Meanwhile, the expression of circadian genes was changed following with amplitude reduced or the peak mRNA level delayed.

**Conclusions:**

Our studies indicated that heperlipidemia altered both the rhythmicity and expression of circadian genes. Diet-induced circadian disruption may affect the process of atherosclerosis and some acute cardiovascular disease.

## Background

In mammals, many behavioral and physiological processes display approximate 24 hour (24-h) rhythms [1] mainly drived by rhythms of transcription of output genes which are not only controlled by the master circadian clock [2-4], localized in the hypothalamic suprachiasmatic nucleus (SCN), but also by the peripheral clock [5-7] in the peripheral tissues, such as heart and liver. As a consequence, many disease symptoms and onset patterns are not randomly distributed within the 24-h period. Especially, most cardiovascular diseases resulting from complications of atherosclerosis, such as stroke and acute myocardial infarction, often occur between 6 a.m and 12 a.m. [8-10], with a peak incidence in the morning. Epidemiological and pathophysiological studies also indicate a causal link between disrupted biological timing and the metabolic syndrome, which is associated with an increased risk of accelerated atherosclerosis and cardiovascular disease [11].

As a metabolic risk factor, hyperlipidemia plays a significant role in the process of atherosclerosis which is characterized by the formation of atheromatous plaques. Many studies have shown circadian genes were involved in regulating liqids metabolism. For example, Rev-erbα-/- mice exhibit a dyslipidemic phenotype with increased very low-density lipoprotein (VLDL) triglyceride levels and apolipoprotein CIII (apoCIII) expression that participates in regulating lipoprotein lipase activity and triglyceride levels [12,13]. Compared to wild-type mice on a high-fat(HF) diet, Clock-/- mice on the same diet showed less triglyceride accumulation in the liver by suppressing expression of *Acsl4 *and *Fabp1 *genes involved in lipid metabolism [14]. However, much less is known about whether and how lipid metabolic process alters the circadian clock. Some metabolic transcription factors have been shown to regulate the expression of circadian genes. For example, Bmal1 transcription is inhibited by REV-ERBa, a transcription factor regulated by adipogenesis [15]. Akira et al, showed that in C57BL/6J mice on HF diets, circadian period of mice was lengthened and the amplitude of circadian gene expression was attenuated, compared to the RC-fed C57BL/6J mice [16].

We previously found that the expression of clock-controlled genes (CCGs, Pai-1, t-PA, TF and ET-1) lost their circadian rhythm in apoE-/- mice fed regular chow (RC) and showed a reverse circadian rhythm in HF-fed apoE-/- mice [17]. Our studies also showed that mammalian apoptosis-associated genes c-myc and p53 exhibited circadian expression in C57BL/6J mice, but the rhythms lost completely in apoE-/- mice [18]. What changes occurred to the expression of circadian genes? In the present study, in the atherosclerotic mice model with hyperlipidemia, we indicated how the expression of circadian genes was changed in suprachiasmatic nucleus (SCN) of the hypothalamus, hearts and livers of atherosclerotic mice and the changed of serum lipid levels. We further analyzed the expression of transcription factors (PPARα, RXRα and Rev-erbα) involved in lipid metabolism and found that hyperlipidemia altered the circadian rhythms in atherosclerotic mice.

## Results

### Analysis of mouse serum lipid

**Figure 1:**
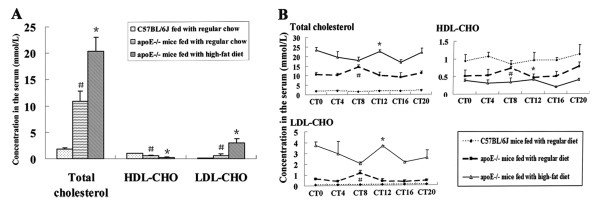
**Concentration of serum cholesterol, LDL-CHO and HDL-CHO in the serum of apoE-/- and C57BL/6J mice**. (A)*P < 0.05 *vs *C57BL/6J mice and apoE-/-mice fed with regular chow; ^#^*P *< 0.01 *vs *C57BL/6J mice fed with regular chow and apoE-/-mice fed with a high-fat diet. (B) *P < 0.05 vs CT16 in HF-fed apoE-/-mice; ^#^*P *< 0.01 vs CT12 in apoE-/-mice on RC

The average levels of total cholesterol and LDL-cholesterol (LDL-CHO) of apoE-/- mice fed with a high-fat diet were higher than those of apoE-/- mice fed with regular chow during a cycle, which is much higher than C57BL/6J wide-type mice. In contrast, the whole level of HDL-CHO of HF-fed apoE-/- mice was the lowest in three group mice, the highest in C57BL/6J wide-type mice (Fig. 1A). The peak concentration of total cholesterol, LDL-CHO and HDL-CHO occurred at CT8 in apoE-/-mice on RC, which delayed four hours in apoE-/-mice on HF diet and reached at CT12, and didn't show in C57BL/6J during a cycle (Fig. 1B).

### Formation of atheromatous plaque in the aorta arch of apoE knock-out mice

**Figure 2:**
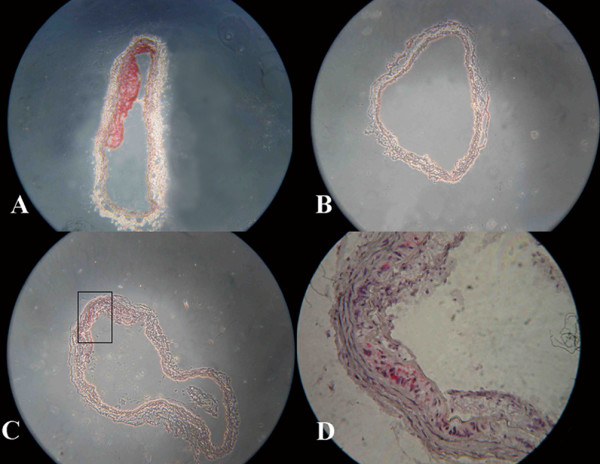
**Atherosclerotic plaques or foam cells showed by oil red O staining in frozen sections of aorta roots of the mice**. (A) Atherosclerotic plaque formed in apoE-/- mouse aortas fed with a high-fat diet, ×100. (B) C57BL/6J mouse, ×100. (C) Foam cells were found in apoE-/- mouse aorta roots, ×100. (D) The rectangle in Fig C shows foam cells under the endothelium, ×400.

Atherosclerotic plaque of aorta root was detected in HF-fed apoE-/-mice for 5 weeks. As shown in Fig. 2, there is no obvious atherosclerotic plaque except for some foam cells deposited under the endothelium in the aorta root of apoE-/- mouse on RC. No lesion was found in the aortic tunica intima in C57BL/6J mouse.

### Diurnal expression patterns of circadian genes in SCN, hearts and livers of apoE-/- atherosclerotic mice

**Figure 3:**
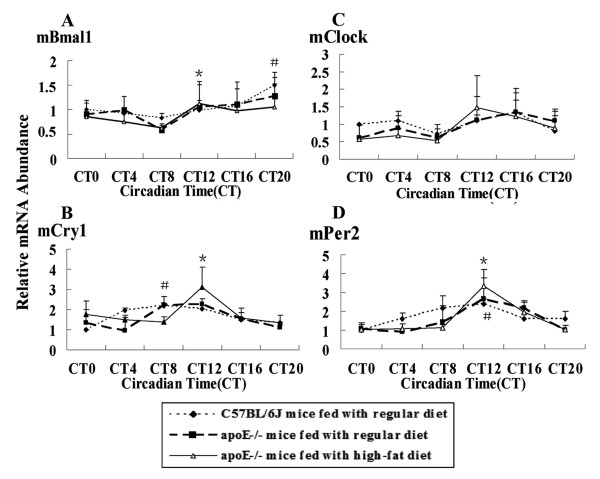
**Diurnal variation of circaidan gene mRNA levels in SCN of C57BL/6J and apoE-/- mice**. The experiment has been repeated three times with similar results. The data from three experiments was normalized to GAPDH mRNA and represented as fold increase over CT0 of C57BL/6J mice. (A) ^#^*P *< 0.05 vs CT8 in C57BL/6J and apoE-/- mice fed with regular chow; *P < 0.05 vs CT8 in HF-fed apoE-/- mice. (B) ^#^*P *< 0.05 vs CT4 in C57BL/6J and apoE-/- mice fed with regular chow; *P < 0.05 vs CT8 in HF-fed apoE-/- mice. (D) ^#^*P *< 0.05 vs CT0 or CT20 in C57BL/6J and apoE-/- mice fed with regular chow; *P < 0.05 vs CT20 in HF-fed apoE-/- mice

Circadian genes in SCN were involved in regulating many kinds of physiological and biological functions. Rhythms were observed except Clock in both C57BL/6J mice and apoE-/- mice, but the amplitude wasn't changed. Bmal1 mRNA level peaked at CT20, the lowest level was at CT8 in C57BL/6J mice Rhythmic expression of Cry1 and Per2 mRNA was similar, the highest mRNA level was found at CT8 and CT12 respectively, and the lowest at CT0 in C57BL/6J mice. In apoE-/- mice on HF diet, the peak mRNA levels of Bmal1, Per2, and Cry1 were showed at CT12, the trough was different (Fig. 3).

**Figure 4:**
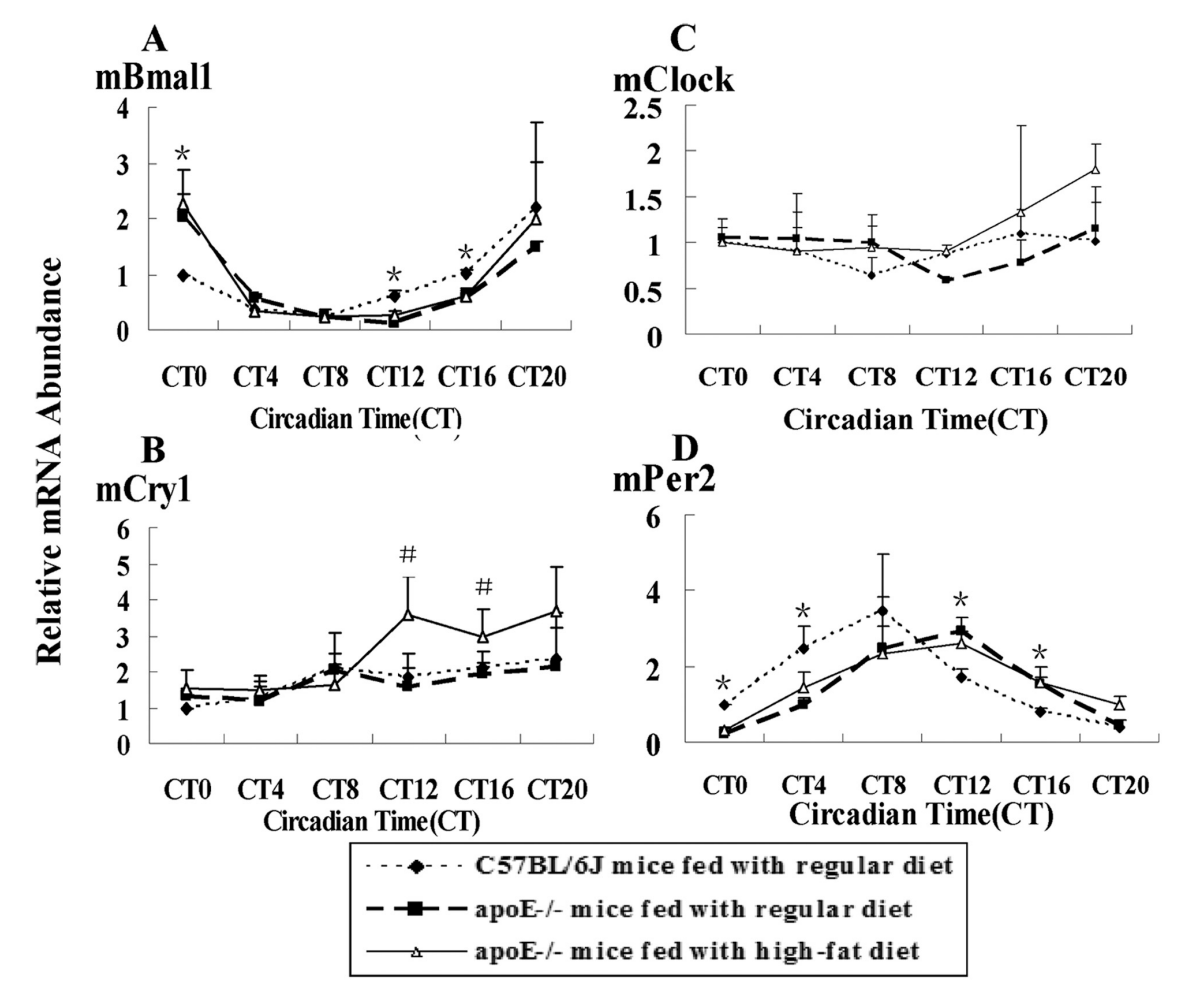
**Diurnal variation of circadian gene mRNA in hearts of C57BL/6J and apoE-/- mice**. The experiment has been repeated three times with similar results. The data from three experiments was normalized to GAPDH mRNA and represented as fold increase over CT0 of C57BL/6J mice. (A)*P < 0.01 vs CT0, CT12 and CT16 in apoE-/- mice fed with regular chow or a high-fat diet. (B)^#^*P *< 0.05 vs CT12 and CT16 in C57BL/6J and apoE-/- mice fed with regular chow. (D) *P < 0.05 vs CT0, CT4, CT12 and CT16 in apoE-/- mice on RC or HF diets

In hearts, the peak mRNA of Bmal1, Per2 and Cry1 was delayed four hours in apoE-/- mice compared to C57BL/6J mice and the amplitude was changed. The peak and trough of Bmal1 mRNA levels were at CT0 and at CT12 in apoE-/- mice, which occurred at CT20 and CT8 in C57BL/6J mice respectively. Similarly, the highest and the lowest Per2 mRNA level were found at CT12 and CT0 in apoE-/- mice, compared to CT8 and CT20 in C57BL/6J mice. There was no significant different in Cry1 mRNA level between C57BL/6J mice and apoE-/- mice on RC. The amplitude of Bmal1 mRNA level was about 2-fold higher at CT0 and about 3-fold lower at CT12 and CT16 in apoE-/-mice than that in C57BL/6J mice. Enhanced expression of Cry1 gene occurred at CT12 and CT16 in HF-fed apoE-/-mice, compared to C57BL/6J mice and apoE-/-mice on RC. Per2 mRNA level was decreased at CT0 and CT4 in apoE-/-mice, in contrast, which was increased at CT12 and CT16, compared to C57BL/6J mice (Fig. 4).

**Figure 5:**
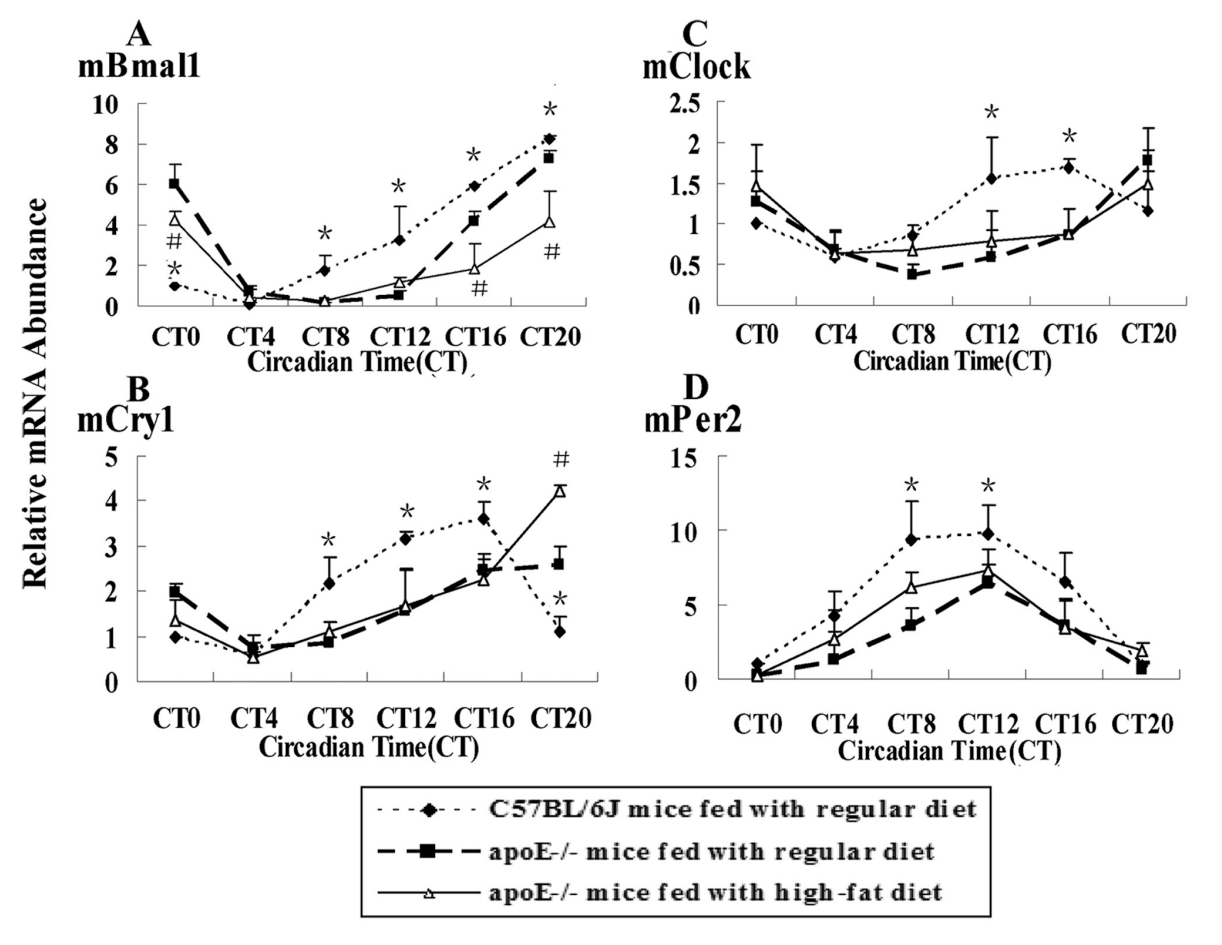
**Diurnal variation of circadian gene mRNA in livers of C57BL/6J and apoE-/- mice**. The experiment has been repeated three times with similar results. The data from three experiments was normalized to GAPDH mRNA and represented as fold increase over CT0 of C57BL/6J mice. (A)*P < 0.05 vs CT0, CT8, CT12 and CT20 in apoE-/- mice on RC or HF diets;^#^*P *< 0.05 vs CT0, CT16 and CT20 in C57BL/6J mice and apoE-/-mice fed with regular chow (B)*P < 0.05 vs CT8, CT12, CT16 and CT20 in apoE-/- mice on RC or HF diets; ^#^*P *< 0.05 vs CT20 in C57BL/6J mice and apoE-/-mice fed with regular chow (C)*P < 0.05 vs CT12 and CT16 in apoE-/- mice on RC or HF diet (D)*P < 0.05 vs CT0, CT8 and CT12 in apoE-/- mice on RC or HF diets

Lipid metabolism processed in liver, circadian gene expression including Clock showed rhythmic oscillation in C57BL/6J or apoE-/- mice. The expression of circadian gene Bmal1 mRNA was decreased at several time points accompanied with the peak delayed. During the whole cycle, Per2 mRNA level was lower in apoE-/-mice than that in C57BL/6J mice. The Cry1 and Clock genes showed a similar expression pattern. The peak mRNA level reached at CT20 with delayed four hours, compared to the peak time point CT16 in C57BL/6J mice (Fig. 5). The expression and rhythmicity of circadian genes in hearts and livers of apoE-/- mice were altered by the hyperlipidemia.

### Diurnal expression profile of transcription factors related with lipid metabolism of apoE-/- atherosclerotic mice

**Figure 6:**
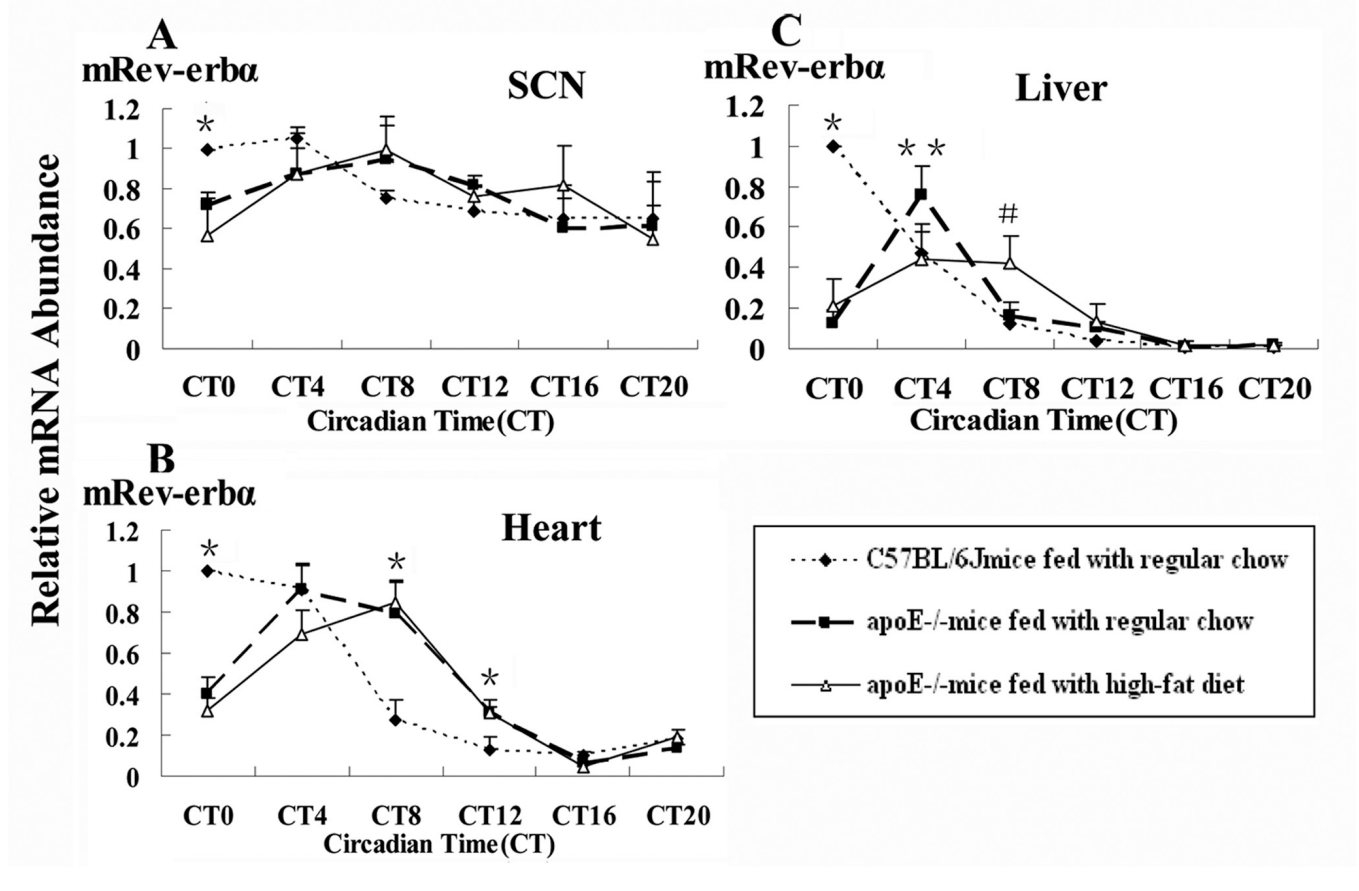
**Diurnal variation of transcription factor Rev-erbα mRNA of C57BL/6J and apoE-/- mice**. The experiment has been repeated three times with similar results. The data from three experiments was normalized to GAPDH mRNA and represented as fold increase over CT0 of C57BL/6J mice. (A)*P < 0.05 vs CT0 in apoE-/- mice on RC or HF diets. (B)*P < 0.05 vs CT0, CT8 and CT12 in apoE-/- mice on RC or HF diets. (C)*P < 0.01 vs CT0 in apoE-/- mice on RC or HF diets; ^#^*P *< 0.05 vs CT8 in C57BL/6J and apoE-/- mice fed with regular chow;**P < 0.05 vs CT4 in C57BL/6J and HF-fed apoE-/- mice

**Figure 7:**
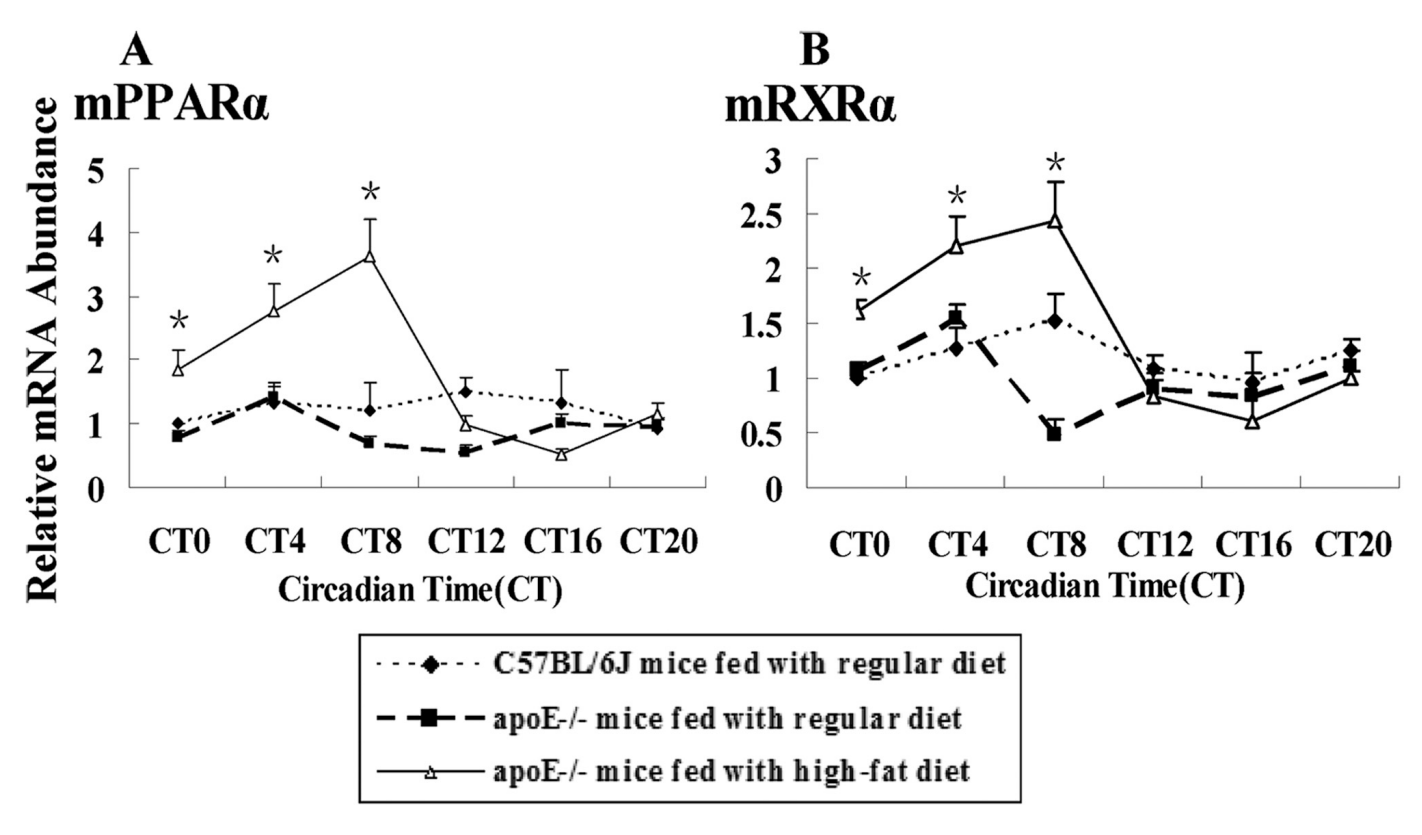
**Diurnal variation of transcription factors PPARα and RXRα mRNA of C57BL/6J and apoE-/- mice**. The experiment has been repeated three times with similar results. The data from three experiments was normalized to GAPDH mRNA and represented as fold increase over CT0 of C57BL/6J mice. (A)*P < 0.01 vs CT0, CT4 and CT8 in C57BL/6J and apoE-/- mice fed with regular chow (B)*P < 0.01 vs CT0, CT4 and CT8 in C57BL/6J and apoE-/- mice fed with regular chow

The similar patterns of clock genes expression alternation induced by hyperlipidemia might be regulated by one or couple of transcription factors binding with their upstream response element. Some of known transcription factors related with lipid metabolism such as Rev-erbα, PPARα and RXRα were detected in SCN, hearts and livers of C57BL/6J or apoE-/- mice. The expression of Rev-erbα was similar in SCN, hearts and livers of C57BL/6J or apoE-/-mice. The peak mRNA level was delayed four hours in apoE-/- mice. At CT0, the amplitude of Rev-erbα expression was decreased about 2-fold to 6-fold in SCN, hearts and livers of apoE-/-mice in succession, compared to that of C57BL/6J mice. These changes occurred only during the light period (Fig. 6). The expression pattern of PPARα and RXRα mRNA wasn't changed in SCN and hearts of C57BL/6J and apoE-/-mice (data not shown), while was daily oscilliation in livers. The mRNA expression of PPARα and RXRα in apoE-/-mice fed with a high-fat diet was increased at CT0, CT4 and CT8, compared to those of C57BL/6J or apoE-/-mice fed with regular chow (Fig. 7). Hyperlipidemia affected the expression of transcription factors.

## Discussion

Onset of acute cardiac events such as myocardial infarction and stroke has a peak in the early morning [19,20]. Although the precise mechanism underlying this phenomenon is still unclear, they are mainly caused by atherosclerosis, which is a chronic vascular disease resulted from complicated causes such as abnormal lipid metabolism and coagulation disorders. In the present study, we used apoE knock-out mice on RC or HF diet as the animal model at early and advanced stage of atherosclerosis, which was identified by serum lipids level analysis and oil red O staining of frozen sections of mice aorta roots that was consistent with our previous results [17,18]. We further investigated whether and how hyperlipidemia, as a risk factor of atherosclerosis, affected the expression of circadian genes.

Our results indicated that the rhythmic profiles of circadian genes were slightly altered in SCN of atherosclerotic mice in comparison with C57BL/6J wild-type mice. The master clock exerted the role of coordinating synchronization of peripheral clock to adapt organism to circumstance. In mammals, light is the most potent entraining signal, with the retinohypothalamic tract (RHT) being the principal retinal pathway through which entraining information reaches the SCN [21,22]. SCN neurons made interaction to entrain the circadian oscillator, which scattered throughout the body. Without the light stimulus, intrinsic circadian clock will be turned off. As shown in our data, the change of diet components had no effect on expression of master circadian genes.

In the peripheral organs, peripheral circadian clock was mainly affected by diet. In the present study, expression of circadian genes in hearts of apoE-/- mice on RC or HF diet presentd difference, where, peak mRNA level was found with four hours delayed in comparison with that of C57BL/6J mice on RC. At the same time, transcription factor Rev-erbα showed four hours delayed, which may regulate the expression and function of circadian genes [23,24]. However, the different expression pattern is that the peak mRNA of circadian genes (Bmal1, Cry1 and Per2) with four hour delayed occurred at the starting of subjective dark period, while transcription factor Rev-erbα at starting of subjective light period in apoE-/-mice, compared to that in C57BL/6J mice, respectively. The concentration of serum lipids showed a peak during a cycle in apoE-/-mice on RC or HF diet. The highest levels of total cholesterol, LDL-CHO and HDL-CHO were observed at CT12 in the starting of dark period in HF-fed apoE-/-mice, which was four hours later than those in RC-fed apoE-/-mice (was at CT8). In C57BL/6J mice, serum lipids level showed no change during the period. The similar expression changes between serum lipids and circadian genes were detected in apoE-/-mice. Disorder of lipid metabolism affected the expression of circadian genes in hearts possibly through transcription factor Rev-erbα which was involved in regulating the lipids metabolism [25].

Our results indicated the peak level of serum lipids delayed in livers of apoE-/- mice, meanwhile, during the subjective light period, the decrease in circulation serum lipids level corresponded with the observed increase of PPARα and RXRα expression levels, transcription factors involved in lipid metabolism. A reported molecular mechanism of the lipid-lowering effect of PPARa was the formation of a PPAR-RXR heterodimer complex, which binds to PPAR response elements (PPREs) in the promoter regions of genes involved in beta-oxidation and lipoprotein/cholesterol transport [26]. In apoE-/- mice, the peak and the trough of circadian gene mRNA levels were observed with four hours delayed, accompanied with mRNA levels decreased at several time points in comparison with C57BL/6J mice. Previous studies demonstrated that several metabolic transcription factors such as PPARα and RXRα have been shown to be involved in regulating Clock, Bmal1 and ClOCK/BMAL1-dependend mRNA expression. For example, the transcription of Per2 is regulated by RXRα, the ligand of PPARα, which regulates lipid and lipoprotein metabolism, and inflammation, major risk factors for atherosclerosis [27,28]. Hyperlipidemia affected the diurnal cycle of circadian genes. The transcriptional factors such as Rev-erbα, PPARα and RXRα may involve in the alternation of circadian gene expression. Altered expression of circadian genes further affected the diurnal expression variation of clock-controlled genes which was involved in atherogenesis and the onset of acute cardiovascular diseases [17,18].

Our study showed that hyperlipidemia and the early and advanced stage of typical atherosclerotic lesions formed in apoE-/- mice fed with regular chow or a high-fat diet. Diurnal variation of serum lipids level affected the expression of circadian genes, which might be mediated by activation of some transcription factors such as PPARα, RXRα and Rev-erbα. This investigation provided new insight into hyperlipidemia-induced circadian disruption may affect the process of atherosclerosis or some acute cardiovascular disease.

## Materials and methods

### Animal model

36 male apoE-/- and 18 male C57BL/6J control (10 weeks postpartum) mice were purchased from Beijing Laboratory Animal Research Center (Beijing, China). ApoE-/- mice were randomly divided into two groups: half of these mice (n = 18) were fed with regular chow, the others (n = 18) were fed with a high-fat diet (containing 0.15% cholesterol and 21% fat). The light period was a 12:12-h light/dark (LD) cycle with light on at 08:00 and light off at 20:00. Mice were adapted to this lighting for 2 weeks and then transferred to a 12:12-h dark/dark (DD) cycle for 3 weeks. Water and food were obtained ad libitum. After DD cycles, mice were sacrificed at four hours internals, starting at CT0 08:00 (CT_circadian time used for assessing biological time without any time cues; CT0 designates the beginning of the subjective day and CT12 20:00 is the subjective night), namely, CT0, CT4, CT8, CT12, CT16, CT20. Mice (n = 3 per group per time point) were sacrificed after deep anesthesia by an intraperitoneal injection of pentobarbital sodium under safe dark red light. Suprachiasmatic nucleus of hypothalamus, hearts and livers were quickly harvested and immediately frozen in liquid nitrogen, and kept at -80°C until use for total RNA extraction. All animal experiments were performed according to the criteria of the Medical Laboratory Animal administrative Committee of Shanghai.

### Analysis of mouse serum lipids

The serum was prepared for serum lipids detection in C57BL/6J mice and apoE-/-mice. The levels of total cholesterol (T-CHO), HDL cholesterol (HDL-CHO) and LDL cholesterol (LDL-CHO) in serum were measured by enzymatic methods [29] using the kits purchased from Rongsheng Biotechnology Company Ltd (Shanghai, China) according to the manufacturer's instructions of detecting.

### Oil Red O staining

The aorta roots were rapidly isolated and the arch of each aorta was removed for frozen sections. The aortic segments were embedded in Tissue-Tek O.T.C compound. Cross-sectional serial sections with a 6-μm thickness were prepared for oil red O staining to show atherosclerotic plaques.

### Total RNA extraction and reverse transcription

Total RNA from suprachiasmatic nucleus, hearts and livers were isolated with Trizol reagent (Invitrogen, Carlsbad, CA), according to the manufacturer's instructions. Two micrograms of total RNA were reversely transcribed and amplified by using the RevertAId™ First Strand cDNA Synthesis kit (Fermentas, Burlington, Canada).

### Real-time PCR

The real-time PCR was carried out by using SYBR-Green Realtime PCR Master Mix with SYBR-Green I (Toyobo, Osaka, Japan) in a total volume of 25 μl. PCR amplifications were performed in a real-time PCR system (Bio-rad) in duplicate. To identify the specificity of the PCR, the PCR products were electrophoresed on ethidium bromide-stained 2% agarose gels, and a single band with expected molecular size for each transcript was confirmed (data not shown). The relative quantification of gene expression was analyzed from the measured threshold cycles (CT) by using the 2- Ct method in the experiment. The data was normalized by determination of the amount of glyceraldehyde-3-phosphate dehydrogenase (GAPDH) mRNA in each sample. Primer sequences of the target genes in the present study were found in Genebank as shown in Table 1.

**Table 1:** The primer Sequences Used for PCR Amplification

Gene	Genebank **Accession No**.	Annealing temperature	Primer sequence 5' to 3'
Per2	NM_011066	58°C	Forward Primer: CAGACTCATGATGACAGAGG Reverse Primer: GAGATGTACAGGATCTTCCC

Bmal1	NM_007489	62°C	Forward Primer: CACTGACTACCAAGAAAGTATG Reverse Primer: ATCCATCTGCTGCCCTGAGA

Clock	NM_007715	58°C	Forward Primer: CTTCCTGGTAACGCGAGAAAG Reverse Primer: TCGAATCTCACTAGCATCTGACT

Cry1	NM_007771	58°C	Forward Primer: CACTGGTTCCGAAAGGGACTC Reverse Primer: CTGAAGCAAAAATCGCCACCT

Rev-Erbα	NM_145434	61°C	Forward Primer: TACATTGGCTCTAGTGGCTCC Reverse Primer: CAGTAGGTGATGGTGGGAAGTA

PPARα	NM_011144	61°C	Forward Primer: TCGGCGAACTATTCGGCTG Reverse Primer: GCACTTGTGAAAACGGCAGT

RXRα	NM_009024	61°C	Forward Primer: CTGCACTCTCCTATCAGCACC Reverse Primer: AGTCCCGAAGCCCAATGTG

GAPDH	BC_083149	62°C	Forward Primer: ACAGCCGCATCTTCTTGTGCAGTA Reverse Primer: GGCCTTGACTGTGCCGTTGAATTT

### Statistical analysis

All data are expressed as means ± SEM. The values for mRNA levels are presented as relative values in all experiments. The oscillation of each gene expression was evaluated by two-way analysis of variance (*ANOVA*) and the post hoc Student's t-test was used to compare the values between the groups at the same CT point, by SPSS 13.0 software. A probability value < 0.05 was considered statistically significant.

## List of abbreviations

SCN: suprachiasmatic nucleus; apoE-/-: apolipoprotein E knock-out; RC: regular chow; HF: high-fat; VLDL: very low density lipoprotein; HDL-CHO: high density lipoprotein cholesterol; LDL-CHO: low density lipoprotein cholesterol; apoCIII: apolipoprotein CIII; Bmal1: Brain and muscle ARNT-like protein 1; PPARα: Peroxisome proliferator-activated receptor alpha; RXRα: Retinoid X receptor alpha; Per2: Period2; Cry1: Cryptochrome 1; Clock: Circadian locomotor output cycles kaput; ET-1: endothelin-1; PAI-1: plasminogen activator inhibitor-1; t-PA: tissue plasminogen activator

## Competing interests

The authors declare that they have no competing interests.

## Authors' contributions

LH (Likun Hou) carried out all aspects of experiments and data analysis, and drafted the manuscript. CL and YH participated in the figure formatting and SC performed the statistical analysis. LH (Luchun Hua) participated in the design of study and proofread manuscript. RQ conceived of the study and performed the experimental instruction. All authors read and approved the final manuscript.
